# TRIMming Down Hormone-Driven Cancers: The Biological Impact of TRIM Proteins on Tumor Development, Progression and Prognostication

**DOI:** 10.3390/cells10061517

**Published:** 2021-06-16

**Authors:** Eleonora Pauletto, Nils Eickhoff, Nuno A. Padrão, Christine Blattner, Wilbert Zwart

**Affiliations:** 1 Institute of Biological and Chemical Systems-Biological Information Processing, Karlsruhe Institute of Technology, PO-Box 3640, 76021 Karlsruhe, Germany; eleonora.pauletto@kit.edu; 2 Division of Oncogenomics, Oncode Institute, The Netherlands Cancer Institute, 1066CX Amsterdam, The Netherlands; n.eickhoff@nki.nl (N.E.); n.padrao@nki.nl (N.A.P.)

**Keywords:** TRIM proteins, hormone-related cancers, E3 ligases, ubiquitin, RING, steroid hormones

## Abstract

The tripartite motif (TRIM) protein family is attracting increasing interest in oncology. As a protein family based on structure rather than function, a plethora of biological activities are described for TRIM proteins, which are implicated in multiple diseases including cancer. With hormone-driven cancers being among the leading causes of cancer-related death, TRIM proteins have been described to portrait tumor suppressive or oncogenic activities in these tumor types. This review describes the biological impact of TRIM proteins in relation to hormone receptor biology, as well as hormone-independent mechanisms that contribute to tumor cell biology in prostate, breast, ovarian and endometrial cancer. Furthermore, we point out common functions of TRIM proteins throughout the group of hormone-driven cancers. An improved understanding of the biological impact of TRIM proteins in cancer may pave the way for improved prognostication and novel therapeutics, ultimately improving cancer care for patients with hormone-driven cancers.

## 1. Introduction

The tripartite motif (TRIM) protein family represents a class of ubiquitously expressed proteins that have different roles in many cellular mechanisms and molecular pathways [1]. The tripartite structure is exclusive to metazoans and has been maintained with few changes throughout evolution. Whilst only a few *TRIM* genes are present in invertebrates (the fruit fly *Drosophila melanogaster* possesses seven *TRIM* genes), a large expansion occurred with the evolution of vertebrates and especially in mammals, with 60 to 70 *TRIM* genes in mouse, rat, dog, cat and cow [2,3]. In humans, there are over 80 known *TRIM* genes [4] that encode proteins that share a similar structure referred to as the RBCC motif. This conserved structure consists of an N-terminal RING (Really Interesting New Gene) domain, adjacent to either one or two cysteine/histidine-rich motifs known as B-boxes (B1 and B2), followed by an alpha-helical coiled-coil region (CC) [5,6]. Eight TRIM proteins stand out for not having a RING domain [4]. In general, TRIMs differ from each other in their C-terminus and in their overall domain structure. According to their C-terminus and domain organization, TRIMs have been classified into eleven subfamilies, C-I to C-XI [7].

Most TRIM proteins have been defined as E3s based on the presence of the RING domain [8], which is responsible for the recruitment of E2 enzymes carrying ubiquitin [9]. Aided by the B boxes to enhance target recognition, the CC region is necessary for the interaction with TRIM family members and other proteins. Indeed, many groups have reported that TRIM proteins form homo- and hetero-interactions (dimers or oligomers) through the interplay between CC regions [10,11,12,13]. The C-terminal domain allows high specificity for substrate recruitment and scaffold and can also have enzymatic activity or chromatin binding capacity [14]. 

## 2. TRIMs: Cellular Processes and Mechanisms

TRIM proteins regulate a high variety of cellular mechanisms, including cell cycle progression, apoptosis, gene expression, chromatin remodeling, signal transduction, metabolism, neurogenesis and stem cell biology (activities comprehensively reviewed in [15,16,17,18,19]). They are also involved in physiological and pathophysiological processes, including development, carcinogenesis and host defense against viral pathogens [15,16,17]. TRIM proteins are often involved in the aforementioned cellular functions as E3 proteins, as part of the so-called ubiquitin cycle [20]. Ubiquitination is a common post-translational modification to control cellular processes, including cell cycle, genome integrity, cell death, inflammatory signaling and defense against pathogens [21]. In this context, E3 ubiquitin ligases, including numerous TRIMs, are necessary for the transfer of ubiquitin from E2 ubiquitin-conjugating enzymes to target proteins in the last step of the ubiquitin cycle [22]. E3s are also responsible for the recognition of target proteins and thus for the efficacy and selectivity of the system [23]. 

Similar to ubiquitination, it has been reported that some TRIMs can control cellular processes by adding ubiquitin-like proteins, such as SUMO [24], ISG15 [25,26] and NEDD8 [27]. 

In addition to the canonical E3 ligase function, TRIM proteins mediate their cellular roles through other mechanisms, including the elimination of misfolded proteins, exerted through different pathways, including autophagy and the endoplasmic reticulum-associated degradation [28,29].

## 3. Steroid Hormone Receptors

The steroid hormone receptor (SHR) family includes the estrogen receptor (ER), androgen receptor (AR), progesterone receptor (PR), glucocorticoid receptor and mineralocorticoid receptor. SHRs are also referred to as type I nuclear receptors, which represent a subfamily of the larger nuclear receptor family that also includes receptors for thyroid hormone, retinoids and nonsteroidal ligands [30,31]. SHRs affect development, cell differentiation, homeostasis, reproduction and metabolism and are associated with several human diseases, including hormone-driven cancers [32]. For example, the role of the ER in breast cancer (BC) cell proliferation, survival and tumorigenesis is well documented and 75% of BC cases are ERα positive [33,34]. Similarly, the AR plays a crucial role in the development and progression of prostate cancer (PC), being a modulator of proliferation, apoptosis, migration and invasion of PC cells [35]. 

SHRs are transcription factors that share a similar and highly conserved structure and possess common functional features centered around the activation function (AF) domains that facilitate transcription [36]. The receptor’s DNA binding domain (DBD) is composed of two zinc finger motifs that bind to steroid responsive elements (SREs). While DBDs are sequence specific, some level of promiscuous association is observed between DBDs and SREs. The specificity of the receptor-DNA interconnection is dictated by the presence of multiple SREs and by the interaction with other facilitating proteins and transcription factors [31,37]. The DBD is connected to the ligand binding domain (LBD) through the hinge region. The latter is a flexible linker that contains a nuclear localization signal, contributes to DNA binding and the transcriptional activities of SHRs, as well as facilitating receptor dimerization and interaction with other proteins [38,39]. The C-terminus encodes the LBD, a well-conserved region among the SHRs [31]. The LBD contains the AF2 with a conserved helix 12 that undergoes extensive conformational changes following ligand binding [40]. 

Steroid hormones possess lipophilic characteristics that allow them to diffuse through cell membranes and to reach the SHRs in the cytoplasm or nucleus [41]. The binding of the ligand to the receptor is facilitated by the heat shock protein 90 (HPS90) complex that maintains the receptor in an optimal conformation for ligand binding [42]. After interaction of the ligand with the steroid hormone receptor, the LBD undergoes a conformational change that results in increased affinity for coregulators, leading to transcription complex formation [31].

## 4. Prostate Cancer

Worldwide, PC is the second most commonly diagnosed male cancer with an incidence of about one in nine men who will develop the disease [43]. The age of diagnosis is mostly above 60 years and the overall 5-year survival for localized lesions is nearly 100%, which decreases to about 30% once metastasis occurs [44]. TRIM proteins affect PC in a variety of ways, acting in a tumor suppressive or oncogenic fashion, reaching from biomarkers for diagnosis [45] to influencing epithelial mesenchymal transition (EMT) [46] and interactions with the AR, which will be further explained below.

### 4.1. TRIMs Involved in Androgen Receptor Biology

Several TRIM proteins are known to interact with the AR including TRIM24, TRIM28 and TRIM68 [47,48,49]. The first two both belong to the class C-VI TRIM proteins, which contain a plant homeodomain and a bromodomain at their C-terminus. Both of these domains are epigenetic readers [50], rendering these TRIM proteins capable of transcriptional regulation, which is also reflected in their alternative names as transcription intermediary factor 1 alpha and beta (TRIM24—TIF1α, TRIM28—TIF1β). In addition to these domains, TRIM24 also contains a LxxLL motif that has been described to interact with the AF2 domain of SHRs including the retinoic acid receptor alpha or the ERα [51,52]. Later it was shown that TRIM24 also interacts with the AR and enhances the transcriptional output of the AR in a ligand- and dose-dependent manner that can be further enhanced through interaction with the histone acetyltransferase TIP60 [47]. In the same study, bromodomain-containing 7 was identified as an interactor of TRIM24, and overexpression of both proteins reduced AR activity compared to TRIM24 overexpression alone. This decrease was less evident when the RING domain of TRIM24 was removed. Interestingly, a mutated TRIM24 lacking the RING domain localized to the cytosol, whereas the full-length protein was detected in the nucleus, which suggests that the above-mentioned effect is due to prevented colocalization of the TRIM24 mutant and the AR. Similar to the effects of TRIM24, TRIM68 displays a dose-dependent stimulation of androgen response and is associated with AR-coactivators like TIP60 and p300 in PC cells. In contrast to TRIM24, the RING domain of TRIM68 is required to enhance the AR’s transcriptional activity, and this effect was abrogated when a proteasomal inhibitor was used. [49]. The above-mentioned observations suggest that substrate ubiquitination and proteasomal degradation are critically involved in TRIM68-mediated regulation of AR activity.

In PC, androgen deprivation therapy (ADT) is prescribed to block AR signaling, but relapse is often inevitable. TRIM24 facilitates proliferation under low androgen levels, which could be a mechanism acting in castration-resistant PC [47,53]. Analysis of the AR-chromatin-binding landscape in LNCaP-abl (ablated) cells, which can proliferate in the absence of androgens, revealed that TRIM24 binds to promoters that are critically relevant for cell proliferation. Genes co-regulated by TRIM24 and AR serve as prognostic markers for poor outcome and these genes are often highly expressed in metastatic PC [53]. This cooperativity is further supported by a study in which TRIM24-chromatin-binding sites were enriched for DNA-consensus motifs for the AR and its pioneer factor FOXA1 [54]. 

Influence on therapy efficacy has been understudied in relation to TRIM proteins in PC, but TRIM24 seems to affect the anti-androgen treatment with bicalutamide. Apart from a pro-proliferative effect of TRIM24 under low androgen levels, TRIM24 also dampens anti-androgen efficiency. One new mechanism how TRIM24 expression can be regulated is via the microRNA miR-137. Analyses of several cell lines and tumor samples showed a negative correlation between TRIM24 and miR-137 expression. As an explanation for higher TRIM24 expression in tumor cells, epigenetic silencing of miR-137 via methylation of promoter proximal CpG islands was suggested [55].

In PC cells, TRIM24 is ubiquitinated and targeted for proteasomal degradation by the ubiquitin ligase speckle-type POZ protein (SPOP) [56]. *SPOP* is mutated in roughly 10% of primary PCs and dominant negative *SPOP* mutations boost AR signaling [57], as the mutations impair SPOP to target coregulators of the AR or the AR itself for proteasomal degradation [58]. However, these results could not explain why TRIM24 levels are higher in PCs that lack *SPOP* mutations. More recently, it was reported that TRIM28 is upregulated in *SPOP* wild-type tumors and protects TRIM24 from SPOP-mediated degradation [54]. The chromatin interactions of TRIM24 and TRIM28 overlapped to nearly 60%, and these sites were no longer occupied by TRIM24 upon TRIM28 knockdown. This study also showed that TRIM28 knockdown reduced overall AR-DNA binding whereas TRIM24 knockdown shifted the AR cistrome to different sites [54]. In general, TRIM28 has a more diverse chromatin distribution than TRIM24, and with mainly zinc finger and CTCF motifs being present in the TRIM28 cistrome, it is more likely to be a repressive transcription factor [59]. Despite this, TRIM28 chromatin interaction profiles also show partial overlap with AR genome-wide binding patterns, but the TRIM28 chromatin interactions were independent of androgen stimulation [48]. Consequently, TRIM28 knockdown decreased colony formation of AR-dependent PC cells and diminished xenograft tumor development [54]. The pathways leading to this phenotype can partially be explained by the TRIM28-AR interactions, as TRIM28 was furthermore reported to facilitate AR activity in a reporter assay [60]. However, other mechanisms, like EMT, DNA damage response and p53 degradation that are associated with TRIM28 in other cancers, could also be of relevance in PC biology (reviewed in [61]). 

In addition to the abovementioned TRIM proteins, TRIM36 is also associated with the AR. *TRIM36* is under direct transcriptional control of the AR and is often highly expressed in PC [62]. However, TRIM36 instead shows tumor suppressive activity in prostate tumors. On the one hand, TRIM36 overexpression increased expression of proapoptotic genes like *BAX* and *TNFSF10*, enhancing apoptotic signaling [63]. On the other hand, TRIM36 knockdown activated MAPK/ERK signaling [64], with increased phosphorylation of ERK. In line with the antiproliferative effects of TRIM36, overexpression of this protein synergized with ADT to reduce growth of LNCaP cells. Since *TRIM36* is a direct AR-responsive gene, the AR/TRIM36 connection may function as a negative feedback mechanism [65]. Nonetheless, TRIM36 may still be lowly expressed in PC despite active AR signaling, which could be mediated through *TRIM36* promoter hypermethylation, as it has been reported in ovarian cancer (OC) and neuroblastoma [66,67]. In a recent preprint, TRIM59 was found to be under direct transcriptional control of the AR as well [68]. In contrast to TRIM36, the activated AR represses *TRIM59* expression. This regulatory mechanism was investigated in the light of treatment-induced neuroendocrine prostate cancer (t-NEPC), where treatment with AR-inhibiting drugs resulted in an upregulation of TRIM59 that showed to be important for xenograft growth in LAPC4 and DU-145 cell lines. Furthermore, the authors linked the growth advantages of TRIM59-overexpressing LNCaP and LAPC4 cells to post-translational downregulation of RB1 and p53 in combination with SOX2 overexpression, which seemed critical for the t-NEPC phenotype [68]. In earlier work, TRIM59 has been associated with androgen-independent growth, which it stimulates by upregulation of general pro-proliferative proteins like cyclins and their respective cyclin-dependent kinases. For TRIM59, it was found that knockdown increased the percentage of cells residing in G0/G1 or S phase as opposed to G2/M, linking TRIM59 with cell cycle progression, which was further supported by reduced protein levels of Cyclin B1 and Cdc2 upon TRIM59 knockdown. Furthermore, TRIM59 is expressed at higher levels in cancerous tissue compared to the adjacent normal tissue, and in vitro and xenograft studies showed that reduced TRIM59 levels diminished cellular proliferation rate and colony formation of PC-3 and DU-145 cells [69].

### 4.2. TRIMs and AR-Independent Signaling Pathways

Even though PC is strongly driven by the AR, many other mechanisms facilitate tumor cell proliferation and enhance metastatic potential, especially in metastatic castration-resistant PC. Known pathways for androgen-independent proliferation are, for example, MAPK signaling, deregulation of cytokines or PI3K/Akt signaling [64].

In addition to the abovementioned perturbations, the altered expression of TRIM proteins can also promote PC independent of AR signaling, such as TRIM44. In a set of knockdown experiments in AR-negative PC-3 cells, proliferation and migration were reduced upon TRIM44 knockdown. These effects were associated with changes in the phosphorylation status of PI3K and Akt, as TRIM44 knockdown decreased the levels of the phosphorylated variants [70]. How these changes are linked to TRIM44 biology and how TRIM44 is regulated in tumors was not investigated.

A cytokine-related pathway that is affected by TRIM proteins is the JAK/STAT signaling axis, which is controlled by TRIM66. Upon TRIM66 knockdown in PC-3 and DU-145 cells, proliferation was decreased, which was attributed to downregulation of STAT2 and IL-2 [71]. However, the exact mechanism remains unknown. As TRIM66 belongs to the TIF1 family, it can be speculated that TRIM66 alters expression of target genes directly. Another member of the TIF1 family is TRIM33, which is reported to act on EMT, as epithelial markers were found downregulated upon shRNA-mediated knockdown of TRIM33. This positive regulation of epithelial proteins could be a possible mechanism for the effect of valproic acid on EMT inhibition in PC, as valproic acid induces TRIM33 expression [72]. Similarly, TRIM16 is lowly expressed in prostate tumors in comparison to healthy prostate epithelial cells and negatively regulates EMT. However, the mechanism of TRIM16 action in PC is different from the other TRIM members described above and has been linked to the EMT-driving transcription factor Snail, which is reduced in cells with higher levels of TRIM16 [46]. 

A more thoroughly studied TRIM that acts independently of the AR is TRIM25. A study in LNCaP and 22Rv1 cells, which was further validated by 22Rv1 xenograft mouse experiments, showed that TRIM25 is essential for the complex formation of p53 and the GTPase-activating protein-binding protein 2 (G3BP2). This complex subsequently recruits the SUMO-ligase RanBP2, resulting in p53 SUMOylation and export from the nucleus [73]. This effect increased migration and proliferation in PC cells as p53-dependent mechanisms like senescence or apoptosis were reduced [74]. An analysis of patient data showed that TRIM25 is upregulated in cancerous lesions and serves as a negative prognostic factor for survival. In line with these findings, knockdown of TRIM25 reduced tumor cell proliferation in vitro and in vivo. Interestingly, the RING domain of TRIM25 was not required for the interaction with G3BP2, as a TRIM25-truncation mutant showed similar effects as compared to the wild-type protein [73]. However, the RING domain is needed for proteasomal degradation of the transcription factor ERG (ETS related gene). ERG drives PC cell proliferation and is often found to be fused to the AR-responsive *TMPRSS2* gene, which leads to aberrant expression and activity of ERG and malignant growth of prostate cells [75]. Interestingly, *TRIM25* is a target gene of ERG and thereby contributes to a negative feedback loop controlling ERG levels. In tumors with overexpressed TRIM25, the deubiquitinase USP9x removes the TRIM25-mediated polyubiquitin chain from ERG to prevent its degradation even when TRIM25 is overexpressed through ERG fusions [76]. 

A unique TRIM protein in regard of function is TRIM19, which is better known as promyelocytic leukemia protein (PML). In the nucleus, TRIM19 oligomerizes and forms spherical nuclear bodies (TRIM19-NB or PML-NB), which can be seen as speckles in immunofluorescence microscopy and have properties of a liquid−liquid phase separation nonmembrane compartment (reviewed in [77]). These TRIM19-NBs are dynamic structures that are involved in several cancer-related mechanisms including alternative lengthening of telomers (ALT), p53-induced senescence, inhibition of Akt signaling or chromatin and SUMOylation dynamics (reviewed in [77,78]). TRIM19 was previously identified as a tumor-suppressive protein in overexpression studies in different PC cell lines [79]. Several translational studies reported that TRIM19 is often misregulated in PC directly, when co-lost with PTEN, forcing a more aggressive growth than the otherwise indolent PTEN-only deficient tumors [80,81,82], or indirectly via deregulation of TRIM19 coregulators. For the latter, it has been shown that proteins that force TRIM19-NB disintegration through post-translational modifications of TRIM19 blunt the otherwise pro-senescence signaling of TRIM19-NBs. Those tumors have higher proliferation rates and worse clinical outcome [83,84,85,86,87]. In relation to AR biology, there is only limited evidence of TRIM19 function, but some results show that TRIM19 can be downregulated upon AR stimulation in CWR22R cells [88]. Moreover, the AR status is an important determinant of TRIM19-driven ALT, which drives AR-independent cell proliferation [89].

## 5. Breast Cancer

With the highest incidence rates of all cancer types in women, BC affects over two million women worldwide [43]. BC is highly heterogeneous in its etiology, with distinct intrinsic subtypes that depend on divergent molecular pathways that drive tumor cell proliferation [90]. TRIM proteins are known to play a role in BC proliferation and progression [91,92,93] and can function as biomarkers for prognostication and disease progression [94,95,96]. 

### 5.1. The Role of TRIMs in BC Tumor Growth and Proliferation

75% of BC cases are ERα-positive [34], and these tumors are generally thought to be critically dependent on the activity of this hormone-driven transcription factor [97]. TRIM24 and TRIM56 were found to interact with ERα, stabilizing its chromatin interactions, thereby enhancing estradiol-stimulated tumor cell proliferation [91,93]. In fact, both TRIMs’ aberrant overexpression in BC is correlated with poor survival of BC patients, and depletion of these TRIMs leads to reduced cell proliferation, as has been shown for tumor-derived BC cells for TRIM24 [91] and MCF7 cells for TRIM56 [93]. 

Despite the clinical success of current BC treatments, around 30% of BC patients will develop metastatic disease [98]. Since targeted treatment options are still lacking for these patients, growing efforts are needed to identify new molecular targets to improve treatment options in the metastatic setting.

Firstly known as estrogen-responsive finger protein, TRIM25 is a downstream transcriptional target of ERα [99,100] and was previously shown to be essential for estrogen-dependent cell proliferation [101]. TRIM25 expression is associated with poor prognosis in BC patients [102]. Furthermore, TRIM25 is associated with BC subtype and is significantly higher expressed in ERα-negative basal BCs. Remarkably however, TRIM25 did not appear under direct transcriptional control of ERα in other studies [103,104]. In fact, overexpression of TRIM25 in ovariectomized athymic mice (absence of estrogens) is still capable of generating tumors. Treatment with an antisense TRIM25 oligonucleotide led to impaired growth of MCF7 tumors implanted in female athymic mice due to an accumulation of 14-3-3σ protein, a G2 cell cycle arrest inducer. It was shown that TRIM25 downregulates 14-3-3σ by acting as an E3 ligase, recruiting the E2 UBCH8 to ubiquitinate the 14-3-3σ protein and lead it to proteasomal degradation [104]. TRIM25 was also reported to regulate key stemness genes including *POU5F1*, *NANOG* and *SOX2*, promoting cell migration and contributing to BC metastasis formation [103,105]. 

TRIM59 was found overexpressed in human breast cancer samples, compared to paired nontumor samples, and was associated with poor prognosis for these BC patients [96]. Furthermore, knockdown of TRIM59 in MCF7 cells reduced cell proliferation in vitro and in vivo, while TRIM59 expression in MDA-MB-231 cells (which express low endogenous levels of TRIM59) enhanced cell proliferation [96]. TRIM28 was also found overexpressed in BC and plays an important role in promoting metastatic disease [92,106,107], as TRIM28 depletion decreased the growth and metastatic potential of tumor xenografts [92]. Moreover, TRIM28 was shown to interact directly with the metastasis-associated transcription factor TWIST1. TRIM28 stabilizes TWIST1, possibly by preventing its ubiquitination [106]. However, as these findings are not yet conclusive, additional research needs to be performed to confirm the biological impact of TRIM28−TWIST1 interaction in metastatic BC progression.

Expression of TRIM13 and TRIM21 is decreased upon BC tumorigenesis and this decreased expression is correlated with poor outcome [95,108]. TRIM21 depletion in ERα-positive BC cell lines MCF7 and T47D increased Snail protein levels and increased cell migration and invasion, with opposite effects observed when TRIM21 was overexpressed [109]. This effect is explained by increased ubiquitination and proteasomal degradation of Snail in the presence of TRIM21. Thus, TRIM21 seems to regulate metastatic transformation of BC cells by regulating Snail protein ubiquitination. TRIM16 levels were also reduced in BC tissue samples compared to the matched normal adjacent breast tissue and its expression is negatively correlated with metastatic progression of BC patients [110]. 

TRIM19 (PML) was found overexpressed in triple-negative BC and correlated with reduced disease-free survival and poor prognosis for patients [111]. TRIM19 inhibition led to impaired cell proliferation and decreased cell cycle progression in triple-negative BC cells [112,113], demonstrating a drug target potential for TRIM19 to treat this subtype of the disease. In ERα-positive BC cell lines (MCF7 and T47D), TRIM19 protein levels were significantly lower as compared to triple-negative BC cell lines [111,113]. Finally, TRIM19 was found to interact with ERα in MCF7 cells and tumors from ERα-positive patient-derived xenografts and ERα-positive human BC tumor samples [114]. Further research would be required to better understand the role of TRIM19 in ERα biology. 

### 5.2. TRIMs in BC Development, Progression and Prognosis

Numerous TRIMs were found to be overexpressed in breast tumors, compared to normal breast tissue: TRIM11, TRIM32, TRIM33, TRIM37, TRIM39, TRIM44, TRIM47 and TRIM63 [94,115,116,117,118,119,120]. TRIM37 overexpression in MCF7 cells led to the silencing of several tumor suppressor genes through H2A monoubiquitination, an epigenetic marker of transcriptional repression. Interestingly, ectopic expression of TRIM37 increased proliferation of MCF10AT cells (a premalignant MCF10A cell line, stably expressing HRAS) in vitro and tumor formation in vivo. These results suggest a role of TRIM37 in BC tumorigenesis by silencing tumor suppressor genes [117]. 

TRIM33 expression was firstly described as decreased in breast tumors, compared to normal breast tissues [121]. However, another study revealed that TRIM33 expression was increased in nearly 40% of BC patients, and its overexpression was related to poor prognosis [122]. Thus, further studies should be performed to fully understand the role of TRIM33 in BC.

## 6. Ovarian Cancer

Ovarian cancer (OC) is a very challenging gynecologic disease, partly due to the late diagnosis that usually occurs, with symptoms appearing at a late stage of cancer progression [123]. Even though its incidence has decreased in recent years, OC is still associated with the highest death rate of all gynecological cancers [124]. 

62% of OCs are considered hormone-responsive cancers, with ERs (both ERα and ERβ) playing a predominant role in their development and progression [125]. While ERα expression is increased in at least a subset of ovarian tumors compared to normal ovarian tissue, ERβ is progressively lost during cancer progression [126].

Different TRIM proteins have been reported to play a role in OC. Two of them (TRIM16 and TRIM50) are considered tumor suppressive [127,128,129,130], while others (TRIM11, TRIM25, TRIM27, TRIM52 and TRIM59) instead promote oncogenesis in this type of cancer [131,132,133,134,135,136]. To date, only TRIM25 has been studied in relation to ERs in OC [137]. 

### 6.1. Tumor Suppressive TRIMs 

TRIM16 was found overexpressed in ER-positive ovarian serous papillary cancers compared to normal tissue [127]. However more recently, TRIM16 was also found to reduce migration and invasion of epithelial OC cells (SKOV3 and OVCAR3) in vitro. In these in vitro models, TRIM16 inhibited EMT by upregulating E-cadherin protein levels (a well-known epithelial marker) and downregulating mesenchymal markers such as N-cadherin and vimentin [128,138]. Overexpression of TRIM16 in SKOV3 cells also downregulated protein levels of the matrix metalloproteases MMP2 and MMP9, which degrade the extracellular matrix, indicating a role of TRIM16 in cancer cell invasion [139]. TRIM16 furthermore inhibited the hedgehog signaling cascade, including sonic hedgehog (Shh), smoothened (SMO), patched (PTCH), and the glioma-associated oncogene homolog-1 (GLI-1) [128]. Shh signaling positively regulates GLI-1 transcription factors in the development of different types of cancer, including OC. After GLI-1 activation through Shh signaling, GLI-1 stimulates EMT in OC cells, enhancing the migration and invasion abilities of SKOV3 cells [129]. A decreased invasive phenotype of BC and hepatocellular carcinoma cells can be caused by the inhibition of MMP2 and MMP9 expression upon suppression of Shh signaling [140,141]. Similarly, TRIM16 may inhibit the migration and invasion abilities of OC cells by downregulating MMP2 and MMP9 through inhibition of the Shh signaling pathway [128].

Gain- and loss-of-function experiments in SKOV3 and A2780 cell lines have also demonstrated that TRIM50 is required for proliferation and migration of OC cells in vitro, as well as for tumor growth in xenograft models in vivo [130]. Low levels of TRIM50 were correlated with higher pathological grades, advanced FIGO (International Federation of Gynecology and Obstetrics) stages and increased lymph node metastasis formation in patients with OC [130]. The tumor suppressive function of TRIM50 is, at least partly, explained by TRIM50-mediated ubiquitination and proteasomal degradation of Src, a proto-oncogene whose expression is correlated with poor prognosis in multiple cancer types [130,142]. 

### 6.2. TRIMs That Promote Proliferation and Metastasis of OC

Multiple TRIM proteins have been reported as being functionally relevant in OC cell proliferation and migration. TRIM27 has been implicated in OC in multiple studies, being higher expressed in cancer samples compared to normal ovarian tissue and higher expressed in cancer cell lines in comparison to untransformed cells [133,134,135]. Furthermore, high expression of TRIM27 in OC was associated with poor prognosis [133,134,135]. Interestingly, downregulation of TRIM27 sensitized the OC cell lines SKOV3 and HEY to carboplatin- or paclitaxel-induced apoptosis, which could be confirmed in mouse xenografts. Taken together, these results imply that TRIM27 could be targeted to enhance the chemosensitivity of OC to carboplatin and paclitaxel [133]. Silencing of TRIM27 in SKOV3 and OVCAR3 cells reduced proliferation and the colony-forming ability of cells, as well as increasing their apoptosis rate (SKOV3 cells), indicating that knockdown of TRIM27 may induce cell cycle arrest and apoptosis [135]. Furthermore, knockdown of TRIM27 increased the number of cells in S-phase of the cell cycle and upregulated the protein levels of p-ATR and p-Chk1 (both required for the S-phase checkpoint [143]). ATR participates in cell cycle arrest by phosphorylating and activating Chk1, which can phosphorylate target proteins such as p53 and trigger cell cycle arrest [144]. Taken together, these results indicate that TRIM27 knockdown could induce S-phase arrest [135]. An increased number of cells in the S-phase could also explain the enhanced sensitivity of OC cells to chemotherapeutics, as cells are generally susceptible to chemotherapy when they are in the S-phase of their cell cycle [145]. Downregulation of TRIM27 also increased phosphorylation of p38 and decreased phosphorylation of Akt in SKOV3 and OVCAR3 cells. p38 and Akt are members of the MAPK and PI3K-Akt pathways, respectively, which are linked to cell proliferation, differentiation and apoptosis [146,147]. The regulation of the abovementioned cascades by TRIM27 suggests a role of TRIM27 in cell proliferation- and apoptosis-related pathways [135]. TRIM27 is under miRNA regulation, as miR-383-5p targets the 3′-UTR of TRIM27 mRNA leading to TRIM27 downregulation. miR-383-5p-mediated silencing of TRIM27 resulted in inhibition of OC cell proliferation, as well as enhanced chemosensitivity to paclitaxel treatment through regulation of the PI3K/Akt pathway [134].

Two independent studies on TRIM59 in OC indicated that TRIM59 is higher expressed in cancerous tissues compared to normal specimens, with high TRIM59 levels being associated with poor prognosis and unfavorable relapse-free survival [131,132]. Both studies observed a reduction in proliferation and migration of OC cells upon silencing of TRIM59. Further analyses of the molecular mechanisms of these regulations identified binding of Annexin A2 to TRIM59 [131]. Annexin A2 was previously described as a prognostic factor in OC [148]. This study also showed that TRIM59 positively regulates Annexin A2 protein expression [131]. On the other hand, TRIM59 was implicated in the FAK/Akt/matrix metalloproteases signaling pathway [132], which enhances metastatic features of tumors, including cell survival, proliferation, migration and invasion [149].

TRIM11 was also found upregulated in OCs compared to adjacent normal tissues [150]. Knockdown of TRIM11 reduced proliferation of A2780 and SKOV3 cells, which was ascribed to decreased Bcl-2 and induction of BAX protein levels upon TRIM11 knockdown. TRIM11 may furthermore be involved in the invasion of OC cells, as downregulation of TRIM11 suppressed MMP2 and MMP9 protein levels [150]. TRIM11 also reduced phosphorylation of ERK and Akt, both of which are involved in the regulation of Bcl-2 and MMP family members [151,152]. By controlling ERK and Akt activity, TRIM11 might lead to deregulation of Bcl-2 and MMP proteins [150,151,152].

Analysis of RNA-seq data from the TCGA database highlighted a significantly higher expression of *TRIM52* in ovarian cancerous tissues compared to normal tissues [136]. Overexpression of TRIM52 in H08910 cells increased cell proliferation, migration and invasion, while knockdown of TRIM52 in SKOV3 and CAOV3 cells reduced cellular migration, invasion and proliferation, yet enhanced apoptosis [136]. TRIM52 was positively correlated with transcript levels of p65, a subunit of the NF-κB complex, in OC specimens. NF-κB downstream effectors were also regulated upon silencing of TRIM52 in SKOV3 and CAOV3 cells. ShRNA-mediated suppression of TRIM52 resulted in downregulation of MMP9, Bcl-2, IL-8 and TNFα protein levels, whilst the protein levels of caspase-3 were upregulated. The opposite phenotype was observed after overexpression of TRIM52 in the HO8910 cell line [136]. 

As described above for PC and BC, TRIM19 (PML) has also been investigated in OC. ShRNA-mediated TRIM19 downregulation decreased cell proliferation and colony formation and increased apoptosis and cleavage of caspase-3 in OV2008 cells [153]. TRIM19 was also shown to accumulate in the nucleus of OV2008 cells upon X-ray irradiation and colocalized with DAXX, a well-established transcriptional regulator [153,154]. TRIM19-DAXX nuclear bodies were described as highly dynamic structures, whose number increased after DNA damage, as shown by the treatment of ES-2 cells with bleomycin [154]. In support of these findings, reduced TRIM19 expression resulted in increased γ-H2AX phosphorylation (an early step in the DNA damage response [155]), whilst TRIM19-DAXX nuclear bodies partially colocalized with γ-H2AX. Altogether, these results indicate that TRIM19, together with DAXX, can have a protective effect on cells after DNA damage [153,154]. 

### 6.3. Other TRIMs

Analogous to BC and PC, TRIM25 has also been associated with OC, being highly expressed in 63% of OC tissues analyzed and associated with a more advanced stage of the disease [137]. 

For many of the TRIM proteins described in this section only SHR-independent mechanisms have been proposed. However, a possible interplay of these TRIM proteins with SHRs cannot be excluded, although no such findings have been reported to date.

## 7. Endometrial Cancer

According to the GLOBOCAN estimates in 2020, endometrial cancer (EC) is the third most common and the fourth most fatal gynecological cancer in women worldwide [43]. The majority of EC cases express high levels of ERα and ERβ, while less than 40% of the cases show positivity for PR (PR-A and PR-B). Typically, EC cases with positivity for ERα, PR-A and PR-B relate to low grade tumors with a favorable disease-specific survival [156].

### TRIMs in EC

Four TRIM proteins have been related to EC: TRIM22, TRIM25, TRIM27 and TRIM44 [157,158,159,160,161,162]. TRIM22 expression was reported to be downregulated in EC samples compared to normal endometrial tissues, and low TRIM22 expression was found to be associated with a high clinical stage of the disease [157]. In the same study, TRIM22 decreased proliferation and migration of KLE, Ishikawa and RL-952 EC cells in vitro and inhibited tumor growth in xenograft models in vivo [157]. These effects are explained by increased levels of the Nucleotide-binding oligomerization domain-containing protein 2 (NOD2) protein upon TRIM22 overexpression in Ishikawa cells [157]. The role of NOD2 in cancer is controversial and may depend on the type of cancer considered. However, NOD2 has been involved in the regulation of numerous cellular pathways including NF-κB-, PI3K- and MAPK-cascades [163]. In the study published by Zhang and coworkers, TRIM22 increased not only NOD2 protein levels but also the protein levels of NF-κB-p65 and IκBα, while their phosphorylation was reduced, resulting in diminished NF-κB signaling [157]. As a parallel effect, TRIM22 also inhibited the activity of NF-κB by preventing the translocation of the p65 subunit into the nucleus. Both mechanisms strongly reduced NF-κB activity and inhibited EC progression [157,164]. In agreement with the findings in BC [165], TRIM22 was under direct transcriptional control of PR-A in EC cells, which was mediated by a direct association of PR-A with the progesterone responsive element in the 5′-upstream region of TRIM22. As progesterone and its analogues are an effective treatment for patients with EC [166], these data suggest that TRIM22 may contribute to the anticancer effects of progesterone in EC [158]

More controversial are the results published for TRIM25 in EC. TRIM25 expression levels were found to be decreased in endometrial tumors relative to healthy tissue [159,161,167]. Moreover, TRIM25 was positively correlated with expression of ERα and PR [159]. Although the data collected in vivo suggested that the effect of TRIM25 on EC could be linked to ERα signaling, in vitro experiments showed that this is not the case. Two independent studies showed a decreased growth rate upon downregulation of TRIM25 in both ERα+ and ERα- EC cell lines, regardless of whether they were treated with estrogens or not [160,161]. Furthermore, TRIM25 downregulation reduced migration of EC cells in the absence of hormone treatment and stopped tumor growth in xenograft mouse models, suggesting that TRIM25 may slow down the transformation process in EC, independently of ERα signaling [160,161].

Different mechanisms have been proposed to explain the role of TRIM25 in the progression of EC. On the one hand, TRIM25 was required for estrogen-dependent activation of vascular endothelial growth factor (VEGF), an important regulator of angiogenesis [160,168]. On the other hand, downregulation of TRIM25 was associated with increased levels of 14-3-3σ protein, both in vitro and in vivo [104]. At the same time, a reporter assay conducted in HEC-1A and Ishikawa cells showed that TRIM25 induced the transcriptional activity of NF-κB [161], whose deregulation is linked to the malignant progression of a large number of cancers, including EC [169]. In agreement with the regulation of NF-κB by TRIM25, TRIM25 knockdown resulted in transcriptional downregulation of NF-κB downstream effectors, including STAT3, STAT5, IFN-γ, IFN-α, interleukin 6 signal transducer (IL6ST) and IL18 [161]. Taken together, TRIM25 is implicated in the progression of EC by enhancing NF-κB signaling, in addition to the downregulation of 14-3-3σ protein. 

In contrast to the TRIMs described above, TRIM44 and TRIM27 have been correlated to a malignant phenotype in EC. TRIM44 expression was found upregulated in EC relative to normal endometrial tissue and correlated to high FIGO stages, high histological grades and lymph node metastases [162]. TRIM44 protein levels were also found associated with poor overall survival of EC patients [162]. Similarly, TRIM27 was correlated to poor prognosis in patients with EC [170]. Furthermore, TRIM27 was also described to facilitate migration and invasion of EC cells in vitro through a process that involves at least the decrease of the protein levels of ITGB1, ITGA2 and ITGA5, three integrins implicated in cancer progression [170,171].

## 8. Discussion 

SHR-driven cancers are amongst the most commonly diagnosed tumor types and are usually sex specific. As for practically all cancers, advances in earlier detection together with improved treatment schemes allowed for decreased mortality while the incidence increased [172]. Today, cancer is still the second leading cause of death worldwide, and roughly 9.9 million patients succumbed to the consequences of cancer in 2020 [43]. In the case of SHR-positive cancers, their ligand-dependent nature provides a unique opportunity for pharmaceutical intervention, and hormonal therapies represent the first and most successful treatments in cancer care. Nonetheless, as the development of resistance to hormonal intervention is common, alternative therapeutic strategies are urgently needed to improve the outcome for these patients. A better understanding of the fundamental biological processes involved in hormone receptor biology would allow a bottom-up approach to link molecular mechanisms to phenotypes and the revelation of possible novel therapeutic avenues in case of endocrine resistance. 

**Figure 1:**
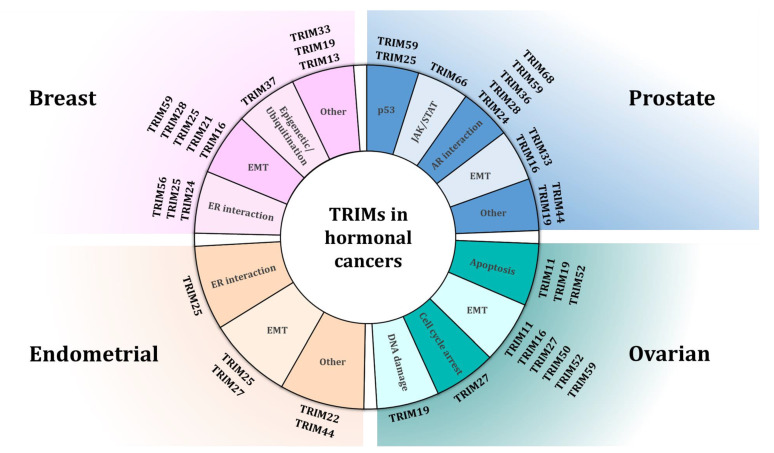
Tripartite motif (TRIM) proteins involved in biological pathways in different hormonal cancer types. TRIM proteins display their effects on the cancer types described, acting on different molecular pathways. Often, for the same TRIM protein, several mechanisms have been proposed.

TRIM proteins are a group of proteins with diverse activities, grouped by structural properties instead of function. As a consequence, TRIMs expose a plethora of molecular functions, some of them being involved in cancer-related processes. Across the board of the investigated cancer subtypes, TRIM proteins affect major pathways specific to cancer (Figure 1). For example, TRIM24 controls AR-driven transcriptional regulation in PC [47,53,56] but also general cancer mechanisms including EMT [128]. As it is feasible to design small molecule inhibitors for TRIM protein family members, as has already been established for TRIM24 [173], therapeutic development programs may be of high importance to increase the druggable space for hormone-driven cancers. 

**Figure 2:**
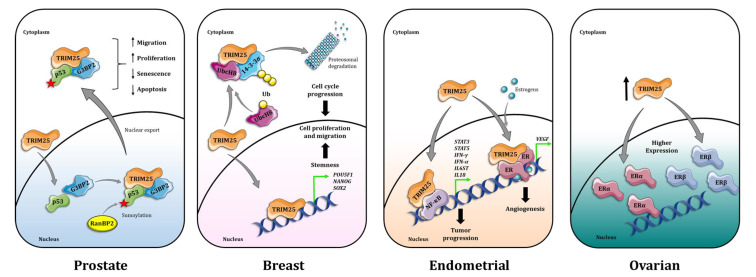
Diversity of TRIM25 action in hormonal cancers. In prostate cancer, TRIM25 binds to p53 and G3BP2, forming a protein complex that facilitates p53 SUMOylation by RanBP2, resulting in p53 nuclear export, increased migration and proliferation of tumor cells while decreasing senescence and apoptosis. In breast cancer, nuclear TRIM25 activates senescence genes, including POUF51, NANOG and SOX2. In addition, TRIM25 acts as an E3 ligase, enabling 14-3-3σ/UBCH8 interaction, leading to 14-3-3σ polyubiquitination and degradation. These two mechanisms of action increase breast cancer cell proliferation and migration. In endometrial cancer, TRIM25 binds to NF-κB, activating transcription of pro-tumorigenic genes, including STAT3, STAT5, IFN-γ, IFN-α, IL6ST and IL18. Upon estrogen signaling, TRIM25 interacts with ERα and activates VEGF gene transcription, enhancing angiogenesis and endometrial tumor progression. In ovarian cancer, TRIM25 is overexpressed relative to normal ovarian tissue, which correlates with increased expression of ERα and ERβ.

Though of clinical and translational interest, many TRIM proteins are understudied in their molecular function and are often only explored in a single tumor type while it remains to be established whether the observations made in one particular setting could reliably be transposed to another. This partial and context-dependent molecular understanding results in the current situation, in which the scientific community is faced with a patchwork of knowledge that needs to be further expanded and unified. Even though some TRIM proteins, like TRIM25, have been studied in numerous cancers and show a similar overall oncogenic function, the underlying mechanisms in which TRIM25 is involved are different, acting as a p53 regulator in PC [73] but as a driver for metastasis in breast cancer [103] (Figure 2). Thus, particular TRIM proteins can play diverse roles in cancer cell biology, which appear to be context-dependent. Therefore, future studies should include extensive molecular and mechanistic analyses in the context of different cancer types, which may reveal both context-dependent as well as more general ubiquitous functions of TRIM proteins. For example, TRIM16 has been associated with EMT in prostate [46], breast [110] and ovarian cancer [128], and an association with Snail regulation has been shown for the first two cancer types. This conserved biological functioning of TRIM16 suggests a commonly shared mode-of-action and positions TRIM16 as a negative regulator of EMT, irrespective of the biological context.

In BC and PC, many of the TRIM proteins act independently of AR or ERα and have been associated with regulator functions for central cancer-related pathways including EMT or apoptosis. These general associations are also seen in other cancers, as reviewed previously [1,4]. However, knowledge from PC on the class C-VI of TRIM proteins and their interaction with AR might be translatable and useful in ERα-driven cancer types. Furthermore, the protein-specific bromodomain present in these proteins allows targeting of these proteins with functional degraders like PROTACS, which has been shown for TRIM24 [174]. This could lead to the discovery of new potential therapeutics, in which the structural similarities between TRIM proteins would represent a major challenge and may come at the cost of a certain level of cross-reactivity within the protein family.

In general, our current understanding on TRIM function in different contexts of oncology, the exact mode of regulation of the TRIMs described in this study and how this deviates between tumor cells relative to normal cells remains far from complete and many studies remain at present associative, lacking deep mechanistical insights. As TRIM proteins act on multiple critical nodes of steroid hormone-dependent carcinogenesis and tumor progression, a deeper mechanistic understanding of TRIM action is critical to fully exploit the clinical potential of TRIMs as drug targets and biomarkers for treatment selection. With the efforts to date, we are still in the early stages of fundamental, pharmaceutical and clinical research on this highly interesting yet complex protein family.
